# Indents

**Published:** 1911-06-15

**Authors:** 


### INDENTS.

☞Brief Articles of Technical or Scientific Value will receive notice and comment. Contributions invited.

*Silver Nitrate Applications.* Dr. Wm. Conrad of St. Louis, advocates the application of this remedy in ten per cent solutions to sensitive areas of the teeth, such as the gingival region and for incipient caries. He applies the silver nitrate solution professionally once a week, but has the patient apply it every day and accompany its application with thorough brushing of the teeth. Applied in this way he claims it will not discolor a live tooth and will stop caries and prevent sensitiveness. Proceedings of Missouri State Dental Society, March 1911, *Western Journal.*

*Hands: To Keep them Soft and White.* Try out the fatty trimmings of mutton by placing them in a dish on the stove (commercial tallow is not suitable). Strain through cheesecloth and beat in sulphur in the proportion of one-half teaspoonful to one-quarter pound of tallow. Rub this thoroughly into the hands at night and wear a pair of kid gloves at least one-half size larger than usually worn, so as not to impede circulation. After a few nights a great improvement will be noticed, and if this simple treatment is used each night the hands will be very soft and white and will never chap.—E. D. Paris, Maine, *Harper's Bazar,* February, 1911.

*To Mix Inlay Investment Compound without Waste.* Place ring on crucible former. Fill not quite full of water and pour quickly into rubber bowl. Sprinkle sufficient investment material to take up water which will be found just enough to invest wax model.—A. G. Loomis, Chicago, Ills., *Review.*

*Fastening Tools in Wooden Handles.* Fill the hole made to receive the tang of the tool with powdered crude sulphur. Heat the tang nearly red hot, and force it into position, holding it firmly until cool. Tools thus fastened seldom give way.—T.

*Cast Saddles.* The use of castings for saddles in connection with all porcelain structures such as diatoric teeth, or any of the detachable pin crowns, or the vulcanite attachments, are of satisfactory construction. You can cast these saddles in metal the thickness of the rolled plate and have a great deal more satisfactory saddle than if it was swaged, for the reason that the saddle is more rigid and less liable to be distorted. One of the great objections with our partial swaged plates is a tendency to an opening of the joint between the vulcanite and the metal owing to the pliability of the metal. The casting has sufficient rigidity with a minimum amount of metal, and that, together with its accuracy, places it far superior to the swaged plate.—F. E. Roach, Chicago, in *Review*.

*A Method to Restore an Imperfect Casting in Aluminum Plate Work.* Some men have the misfortune sometimes of having an imperfect cast in their aluminum work. If you will be careful in separating your flask after casting, and find you have an imperfect cast while the cast is yet on the model, take a small piece of the material, place over the defect, then heat with a needle-point flame from your blow-pipe for a radius of half an inch around the parts; when the metal and the surrounding parts are in a partially molten state iron all together with a suitable round-end spatula. You have then saved your cast.—Dr. J. F. Gatts, *Summary*.

*Restoration of Badly Broken Down Roots* When pins can be placed parallel in the roots to be restored, it is easy, but I find that such cannot always be accomplished

*with Acolite.* especially if the tooth has been so long decayed that the roots are now separated. In such a case prepare root and insert pin through disk of 36 gold, burnishing to place on root and strengthen with solder, making separate cap and pin for each root, and bending the portion of pin left above cap to be parallel with the others, cutting partly through with saw if necessary to bend the more easily, then soldering again. With pins in position on roots secure a model of restoration desired in inlay wax, invest cast and set as one does a Davis crown. The holes left by pins may need slightly enlarging before restoration will go to place, as the force of the metal in casting will cause the investment at that point to slightly crumble. A narrow metal band approximately the size and shape of cast desired may be used while taking the impression in the wax to prevent the spreading of wax at the gum margin when the patient bites on it.—Dr. Kilbourne in *Summary.*

*The proper Application of the Saliva Ejector.* It is entirely unnecessary to subject a patient to a prolonged and tedious sitting with the saliva-ejector under the rubber-dam and cramped upon the lip. After adjusting the dam, cut an opening with a curved crown-shears, remote from the field of operation, and insert ejector through this opening.

You will be surprised at the comfort you have rendered the patient, and, above all, it overcomes the closing of the apertures in the injector—a very frequent and annoying affair to both patient and operator. Do this with your next case and watch the change.—Bernard Bramm, Chicago, *Dental Review.*

